# Sociodemographic and Clinical Factors Associated With Response to 1-Year Postarthroplasty Patient-Reported Outcome Measures

**DOI:** 10.1016/j.artd.2025.101804

**Published:** 2026-03-30

**Authors:** Fatoumata Sylla, Brocha Z. Stern, Martha Burla, Linda I. Suleiman, Patricia D. Franklin

## Introduction

Patient-reported outcome measures (PROMs) have emerged as an essential tool for evaluating the effectiveness of medical interventions from the patient's perspective. By systematically capturing the patient’s voice, PROMs provide invaluable insights that can facilitate shared decision-making between patients and clinicians and improve clinical outcomes [1]. Health systems can also use PROMs for a more patient-centered approach to assessing performance, a process that has already been initiated by the Centers for Medicare & Medicaid Services (CMS) in orthopaedic surgery [2]. Specifically, CMS has mandated a patient-reported outcome-based performance measure for hospital quality reporting for inpatient elective total hip (THA) and knee arthroplasty (TKA) procedures, with expanded implementation announced for hospital outpatient departments and ambulatory surgery centers [3].To avoid financial penalties from CMS, hospitals must report paired preoperative and 1-year postoperative data on a minimum of 50% of cases [3]. These current mandates illustrate CMS’s increasing emphasis on value-based care. It is likely that future requirements will involve even higher reporting thresholds and broader adoption of PROM metrics, further strengthening the focus on patient-centered outcomes.

To meet these evolving requirements, representative real-world data, including PROMs, are necessary for meaningful performance measurement. While 50% is the minimum PROM response rate required under the CMS quality reporting mandate, the International Society of Arthroplasty Registries recommends achieving a minimum response rate of 60% or higher to ensure robust data [4]. However, longitudinal follow-up for PROMs remains a challenge. For example, the 2024 American Joint Replacement Registry report indicates linked preoperative and 1-year PROM data on only 24%-30% of cases [5]. Studies have highlighted that certain sociodemographic groups, including racial and ethnic minorities, are less likely to complete PROMs overall [6,7]. Limited options for response methods, including reliance on independent completion via patient portals, may specifically limit response rates in diverse populations [8]. However, such automated digital solutions may especially be relied on for follow-up collection when patients are no longer seen in the clinic. A better understanding of patient subgroups that may require additional support for PROM completion can help with targeted resource allocation. Therefore, this study aimed to identify sociodemographic and clinical factors associated with 1-year PROM completion among THA and TKA patients, including whether surveys were completed independently or following staff contact. As a secondary aim, we compared 1-year PROM scores by the response method.

## Material and methods

### Design and procedures

This retrospective secondary analysis used data from a multicenter pragmatic clinical trial (“Arthritis care through Shared Knowledge” [A.S.K.]) that assessed patient decision quality when using a personalized PROM-based decision report (*masked for review*) [9]. The study procedures were approved by the (*masked for review*) institutional review board (*masked for review*), with all participants providing informed consent. The parent trial included English- and Spanish-speaking adults who were seeking care for hip or knee osteoarthritis at participating A.S.K. surgeons across several academic and community practices in the United States. Patients completed the Hip Disability and Osteoarthritis Outcome Score (HOOS-12) or the Knee Injury and Osteoarthritis Outcome Score (KOOS-12) [10,11], which provide estimates of joint-related pain, function, and quality of life. Higher HOOS-12 or KOOS-12 scores indicate less pain and better function. Patients also completed the Veterans RAND 12-Item Health Survey (VR-12), which provides a mental component summary (MCS) score and a physical component summary score, with higher scores indicating better global mental or physical health [12].

These PROMs were collected before the orthopaedic surgery consultation and at 6 months and 1-year follow-ups. Patients scheduled for a consultation were enrolled by staff in the survey platform and completed the preconsultation demographic and clinical assessment (including PROMs) from home -via an emailed survey link or telephone-or by tablet in the clinic prior to onset of COVID-19 [13]. Follow-up PROMs were completed using a Health Insurance Portability and Accountability Act-compliant survey platform (DatStat with migration to Redcap during the study). The participants' email invitation uses a secure personalized survey link through which they can access and complete the PROM from home without a specific log-in password. For follow-up PROM completion, up to 4 email invitations were sent for independent electronic completion via DatStat or REDCap; however, research coordinators could send additional email reminders. Patients who did not respond were subsequently contacted via telephone up to 3 times by centralized research staff to remind them to complete the PROMs via the link in their email invitation. Staff left voice mail messages when the patient did not answer calls. When telephone contact was made and patients required assistance with electronic PROM completion beyond a reminder, staff provided such support (eg, reading the questions to a patient and recording their responses). The 1-year electronic PROM invitations were first sent via email 330 days after the consultation. Participants were added to a research coordinator call list at 365 days and remained eligible for follow-up through 470 days. This collection window provided flexibility while maintaining alignment with the intended 1-year follow-up timeframe. Across all A.S.K. trial respondents who completed a 1-year PROM, the median days from initial appointment to completion was 367 (interquartile range 335-397).

### Participants and variables of interest

**Figure 1:**
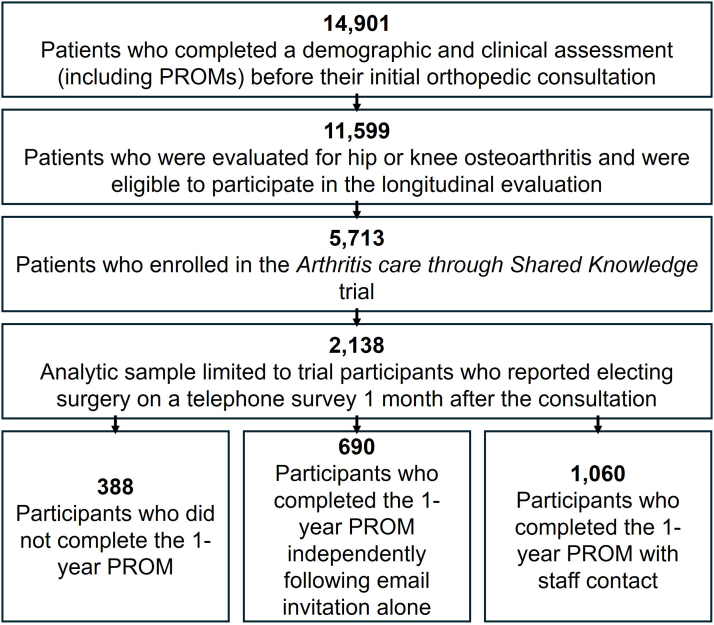
Flowchart of creation of analytic sample.

For this analysis, the sample was limited to A.S.K. patients who elected surgery within 1 month of the orthopaedic surgery consultation (Fig. 1 shows sampling flow). Sociodemographic characteristics included self-reported age, sex, race, ethnicity, marital status, highest education, perceived health literacy, and health insurance status. Health literacy was assessed using the Single-Item Literacy Screener about confidence to complete medical forms and was categorized as limited vs adequate [14]. Health insurance was categorized as private only, Medicare, Medicaid (including dual Medicare/Medicaid eligibility), and other/none/missing. Clinical characteristics included body mass index, self-reported medical comorbidities via the Charlson Comorbidity Index, low back pain severity via the Oswestry low back pain item, number of other painful hip/knee joints, cigarette smoker status, preoperative VR-12 MCS scores, and operative joint (hip vs knee). We also extracted the preoperative HOOS-12 or KOOS-12 Pain and Function scores. Additionally, we identified whether or not patients reported discussing the personalized decision report with their surgeon at their consultation.

The primary outcomes were (a) a response (vs no response) to the 1-year HOOS/KOOS-12 and (b) whether the 1-year HOOS/KOOS-12 was completed independently following email invitation alone or required staff contact for completion. Those who responded at 12 months via any method were categorized into the “response” group while those who never responded were categorized into the “no response” group. Among respondents, patients who responded to the email invitation with no additional staff contact were categorized into the “email invitation alone” response group, and those who required contact with a staff member to complete were categorized into the “staff contact” response group. The email invitation alone condition is a proxy for automated electronic solutions often used for longitudinal follow-up.

### Data analyses

Sociodemographic and clinical variables were summarized using means and standard deviations for continuous variables and frequencies and percentages for categorical variables. Univariable analyses were used to compare characteristics between the response and no response groups and between the email invitation alone and staff contact groups using chi-square tests for categorical variables or independent t-tests for continuous variables. We constructed a multivariable binary logistic regression model with response vs no response as the outcome. All of the previously specified covariates were included except for discussion of the report with the surgeon. Preoperative HOOS-12 and KOOS-12 scores were aggregated into HOOS/KOOS-12 Pain and HOOS/KOOS-12 Function for this model. The same model was also constructed with response following email invitation alone (vs with staff contact) as the outcome. We report adjusted odds ratios (ORs) and 95% confidence intervals (CIs) for multivariable models. As a secondary analysis, we compared 1-year HOOS-12 or KOOS-12 Pain and Function scores based on the response method using independent t-tests. We also qualitatively examined differences in postoperative PROM scores between the email invitation alone and the full response group to see if mean patient profile scores changed with the inclusion of additional participants from the staff contact group. The alpha level was set at a 2-tailed *P* value of <0.05 for all analyses.

## Results

### Response rates

The total sample included 2138 participants (58.4% female, 90.5% White), with a mean age of 66.8 (standard deviation 9.0). The 1-year response rate was 32.3% (n = 690) when response was limited to independent completion following email invitation alone but increased to 81.9% (n = 1750) when response rate was calculated across the independent completion and staff contact groups combined.

### Response vs No response

For sociodemographic factors, higher unadjusted response rates were noted in females compared to males (*P =* .020), those who were married vs not (*P =* .008), and those who were college graduates compared to not (*P* < .001). Lower unadjusted response rates were noted in those who identified as Hispanic/Latino (*P =* .005), with limited vs adequate health literacy (*P =* .016), and who had Medicaid vs other insurances (*P =* .001; Table 1). For clinical factors, higher unadjusted response rates were noted in those with a lower medical comorbidity burden (*P =* .002), nonsmokers vs smokers (*P* < .001), and those with slightly better preoperative mental health (*P* < .001). Similarly, patients with higher (ie, better) preoperative HOOS/KOOS-12 Pain scores were more likely to respond (hip: *P =* .001; knee: *P =* .008). Knee patients who responded also had higher (ie, better) preoperative KOOS-12 Function scores than those who did not respond (*P =* .006; Table 1).

**Table 1:** Univariable comparisons of sociodemographic and clinical characteristics by 1-yr response and response method.

Variable	Level	Response	No response	*P* value	Email invitation alone	Staff contact	*P* value
		n (%)	n (%)		n (%)	n (%)	
Age	*(mean, SD)*	66.9 (8.7)	65.6 (9.8)	.081	66.8 (8.6)	67.0 (8.8)	.620
Sex	Male	708 (40.5)	182 (46.9)	**.020**	294 (42.6)	414 (39.1)	.139
	Female	1042 (59.5)	206 (53.1)		396 (57.4)	646 (60.9)	
Race	White	1593 (91.0)	341 (87.9)	.087	643 (93.2)	950 (89.6)	**.016**
	Black or African American	103 (5.9)	27 (7.0)		27 (3.9)	76 (7.2)	
	Other/Missing	54 (3.1)	20 (5.2)		20 (2.9)	34 (3.2)	
Ethnicity	Not Hispanic or Latino	1727 (98.7)	375 (96.6)	**.005**	681 (98.7)	1046 (98.7)	.977
	Hispanic/Latino	23 (1.3)	13 (3.4)		9 (1.3)	14 (1.3)	
Marital Status	Married	1157 (66.6)	230 (59.4)	**.008**	484 (70.6)	673 (64.0)	**.004**
	Unmarried	581 (33.4)	157 (40.6)		202 (29.5)	379 (36.0)	
Highest Education	College Graduate	876 (50.4)	148 (38.2)	**<.001**	390 (56.9)	486 (46.2)	**<.001**
	Trade/Technical School or Some College	451 (26.0)	120 (31.0)		173 (25.2)	278 (26.5)	
	High School or Less	372 (21.4)	109 (28.2)		111 (16.2)	261 (24.8)	
	Other/Missing	38 (2.2)	10 (2.6)		12 (1.8)	26 (2.5)	
Health Literacy	Limited	144 (8.3)	47 (12.1)	**.016**	50 (7.3)	94 (8.9)	.227
	Adequate	1598 (91.7)	341 (87.9)		637 (92.7)	961 (91.1)	
Health Insurance	Private	632 (36.1)	132 (34.0)	**.001**	267 (38.7)	365 (34.4)	**.029**
	Medicare	850 (48.6)	172 (44.3)		338 (49.0)	512 (48.3)	
	Medicaid	97 (5.5)	42 (10.8)		29 (4.2)	68 (6.4)	
	Other/Missing/None	171 (9.8)	42 (10.8)		56 (8.1)	115 (10.9)	
Body Mass Index	*(mean, SD)*	30.5 (5.8)	30.8 (5.9)	.409	30.3 (5.7)	30.6 (5.8)	.270
Medical Comorbidities	0	852 (43.8)	161 (41.5)	**.002**	365 (53.1)	487 (46.1)	**.013**
	1	538 (27.7)	133 (34.3)		191 (27.8)	347 (32.8)	
	2-5	551 (28.3)	89 (22.9)		132 (19.2)	219 (20.7)	
	6-20	4 (0.2)	5 (1.3)		0 (0.0)	4 (0.38)	
Low Back Pain	None/Mild	1241 (71.1)	260 (67.0)	.109	500 (72.8)	741 (70.0)	.217
	Moderate/Severe	504 (28.9)	128 (33.0)		187 (27.2)	317 (30.0)	
Other Painful Joints	0	818 (46.9)	179 (46.1)	.792	329 (47.9)	489 (46.3)	.382
	1	670 (38.4)	147 (37.9)		269 (39.2)	401 (38.0)	
	2	173 (10.4)	39 (10.1)		63 (9.2)	110 (10.4)	
	3	82 (4.7)	23 (5.9)		26 (3.8)	56 (5.3)	
Cigarette Smoker	Yes	116 (6.7)	52 (13.4)	**<.001**	37 (5.4)	79 (7.5)	.085
	No	1625 (93.3)	336 (86.6)		650 (94.6)	975 (92.5)	
Preoperative MCS	*(mean, SD)*	56.6 (11.0)	54.0 (11.7)	**<.001**	57.6 (10.2)	56.0 (11.4)	**.002**
Operative Joint	Hip	812 (46.4)	161 (41.5)	.079	335 (48.6)	477 (45.0)	.146
	Knee	938 (53.6)	227 (58.5)		355 (51.5)	583 (55.0)	
Hip Patients: Preoperative HOOS-12 Pain	*(mean, SD)*	39.3 (16.3)	34.7 (15.4)	**.001**	41.7 (15.8)	37.6 (16.5)	**<.001**
Knee Patients: Preoperative KOOS-12 Pain	*(mean, SD)*	41.0 (15.8)	37.5 (17.6)	**.008**	41.5 (16.1)	40.6 (15.5)	.406
Hip Patients: Preoperative HOOS-12 Function	*(mean, SD)*	45.9 (19.3)	42.6 (19.1)	.051	48.3 (19.0)	44.3 (19.3)	**.005**
Knee Patients: Preoperative KOOS-12 Function	*(mean, SD)*	47.1 (19.1)	42.9 (20.9)	**.006**	48.5 (18.8)	46.3 (19.2)	.089
Used Personalized Decision Report with Surgeon	Yes	699 (40.3)	143 (37.2)	.262	319 (47.0)	380 (36.1)	**<.001**
	No	1034 (59.7)	241 (62.8)		360 (53.0)	674 (63.9)	

*P*-values reflect chi-square tests for categorical variables and independent t-tests for continuous variables.

Bolded *P* values indicate statistical significance (*P* < .05).

SD, standard deviation.

After adjusting for other factors, differences persisted in sex, education, smoking status, preoperative mental health scores, and hip/knee pain (Table 2). Specifically, females had a higher likelihood of responding compared to males (OR 1.48, *P =* .002). Those who had completed trade/technical school or some college (OR 0.71, *P =* .019) and those who had completed high school or less (OR 0.71, *P =* .027) were less likely to respond compared to those with higher levels of education. Cigarette smokers were less likely to respond compared to nonsmokers (OR 0.60, *P =* .009). Higher preoperative VR-12 MCS scores and higher preoperative HOOS/KOOS-12 Pain scores were associated with an increased likelihood of responding (VR-12 MCS: OR 1.01, *P =* .011; HOOS/KOOS-12 Pain: OR 1.01, *P* = .036). Additionally, those with 1 vs 0 other painful joints were more likely to respond (OR 1.31, *P =* .048), and patients undergoing knee vs hip surgery were less likely to respond to the 1-year PROM (OR 0.78, *P =* .040; Table 2). There were no longer significant associations for ethnicity when adjusting for other factors.

**Table 2:** Adjusted associations between sociodemographic and clinical characteristics and the outcomes of 1-y response and response method.

Variable	Level	Response vs No response		Email invitation alone vs Staff contact	
		OR (95% CI)	*P* value	Or (95% CI)	*P* value
Age	-	1.00 (0.99-1.02)	.773	1.00 (0.98-1.01)	.588
Sex	Female vs Male	1.48 (1.16-1.89)	**.002**	0.96 (0.78-1.20)	.739
Race	Black or African American vs White	1.11 (0.68-1.79)	.681	0.60 (0.37-0.97)	**.036**
	Other/Missing vs White	0.68 (0.37-1.22)	.197	0.87 (0.47-1.60)	.650
Ethnicity	Hispanic or Latino vs Not	0.84 (0.36-1.95)	.693	1.46 (0.59-3.65)	.416
Marital Status	Unmarried vs Married	0.79 (0.62-1.02)	.075	0.81 (0.65-1.02)	.075
Highest Education	Trade/Technical School or Some College vs College Graduate	0.71 (0.53-0.94)	**.019**	0.83 (0.65-1.06)	.131
	High School or Less vs College Graduate	0.71 (0.52-0.96)	**.027**	0.59 (0.45-0.78)	**<.001**
	Other vs College Graduate	0.59 (0.28-1.25)	.168	0.45 (0.20-1.02)	.054
Health Literacy	Adequate vs Limited	1.01 (0.68-1.50)	.960	0.86 (0.58-1.27)	.450
Health Insurance	Medicare vs Private	1.04 (0.76-1.43)	.798	1.00 (0.76-1.32)	.978
	Medicaid vs Private	0.83 (0.52-1.33)	.444	0.76 (0.46-1.26)	.284
	Other/Missing/None vs Private	0.94 (0.62-1.42)	.772	0.73 (0.50-1.08)	.119
Body Mass Index	-	1.01 (0.99-1.03)	.547	1.01 (0.99-1.03)	.453
Medical Comorbidities	1 vs 0	0.79 (0.60-1.03)	.082	0.80 (0.63-1.01)	.060
	2 vs 0	0.85 (0.59-1.22)	.380	1.05 (0.77-1.45)	.741
	3+ vs 0	0.66 (0.43-1.02)	.060	0.61 (0.39-0.95)	**.027**
Low Back Pain	Moderate/Severe vs None/Mild	0.99 (0.76-1.30)	.946	0.98 (0.77-1.25)	.872
Other Painful Joints	1 vs 0	1.31 (1.00-1.70)	**.048**	1.11 (0.88-1.39)	.370
	2 vs 0	1.36 (0.89-2.08)	.158	1.02 (0.70-1.49)	.901
	3 vs 0	1.31 (0.75-2.29)	.344	0.92 (0.54-1.57)	.757
Cigarette Smoker	Yes vs No	0.60 (0.41-0.88)	**.009**	0.82 (0.53-1.26)	.365
Preoperative MCS	-	1.01 (1.00-1.03)	**.011**	1.01 (1.00-1.02)	.091
Operative Joint	Knee vs Hip	0.78 (0.61-0.99)	**.040**	0.83 (0.67-1.02)	.073
Preoperative HOOS/KOOS-12 Pain	-	1.01 (1.00-1.02)	**.036**	1.00 (0.99-1.01)	.469
Preoperative HOOS/KOOS-12 Function	-	1.00 (0.99-1.01)	.760	1.00 (0.99-1.01)	.743
Constant		1.27 (0.29-5.53)	.746	0.66 (0.17-2.56)	.545

Bolded *P* values indicate statistical significance (*P* < .05).

### Email invitation alone vs staff contact

Of the 1750 participants who responded to the PROM at 12 months, 39.4% were in the email invitation alone group (*n* = 690). For sociodemographic factors, the email invitation alone group included a larger unadjusted proportion of White patients compared to those in other racial categories (*P =*.016), those who were married vs not (*P =* .004), and those who were college graduates compared to those with less education (*P* < .001). Additionally, those with Medicaid insurance had a larger proportion in the staff contact vs email invitation alone group (*P =* .029; Table 1). For clinical factors, patients with a lower medical comorbidity burden were more likely to be in the email invitation alone group (*P =* .013). Additionally, those in the email invitation alone group had slightly higher preoperative mental health scores (*P =* .002). Hip patients in the email invitation alone group also had higher (ie, better) preoperative HOOS-12 Pain (*P* < .001) and Function scores (*P =* .005). Those who used the personalized report of pain and function with their surgeon were also more likely to respond independently following email invitation alone (*P* < .001; Table 1).

After adjusting for other factors, differences persisted in race, education status, and medical comorbidities. Specifically, Black or African American (vs White) patients were less likely to respond following email invitation alone (OR 0.60, *P =* .036), and patients with a high school education or less (vs college graduates) were also less likely to respond following email invitation alone (OR 0.59, *P* < .001). Additionally, for medical comorbidities, patients with 3 or more comorbidities were less likely to respond following email invitation alone compared to those with no comorbidities (OR 0.61, *P =* .027; Table 2).

### Comparisons of postoperative PROM scores by response method

Postoperative 1-year PROM scores are summarized in Table 3. There were no significant differences in 1-year scores between those who responded following email invitation alone vs staff contact. Qualitatively, the mean 1-year scores for those who responded via email invitation alone vs the mean scores across all respondents were similar, with a maximum of a 1.4-point difference for the 1-year KOOS-12 Function scores.

**Table 3:** Comparing 1-y patient-reported outcome measure scores by the response method.

Variable	Email invitation alone Mean (SD)	Staff contact Mean (SD)	*P* value	All respondents Mean (SD)
Hip Patients				
1-Y HOOS-12 Pain	87.7 (17.3)	86.6 (19.3)	.410	87.1 (18.5)
1-Y HOOS-12 Function	88.7 (16.8)	88.1 (17.7)	.599	88.4 (17.3)
Knee Patients				
1-Y KOOS-12 Pain	77.1 (21.8)	75.8 (23.5)	.390	76.3 (22.9)
1-Y KOOS-12 Function	79.5 (20.6)	77.3 (23.3)	.132	78.1 (22.3)

*P* values reflect independent t-tests.

SD, standard deviation.

## Discussion

This study identified that 1-year postoperative PROM response and response methods in patients undergoing THA or TKA were associated with several patient factors, including sex, race, and education. While other studies have examined associations between sociodemographic factors and response rates, these findings add to the literature by specifically focusing on responses to 1-year postoperative PROMs in elective THA and TKA to align with the CMS quality measure. Including staff contact was necessary to increase the 1-year response rate above the CMS threshold of 50%. Staff contact also increased representation among subgroups of Black or African American patients and individuals with a high school education or less, suggesting the need for targeted interventions to address disparities in PROM response.

In the current study, higher response rates were observed among females, similar to past research [6,15]. Black or African American patients were less likely than White patients to complete PROMs following email invitation alone, but there were no longer significant differences in response when staff contact was added, suggesting barriers specifically to independent electronic completion. Past research suggests overall mixed findings for associations between race and response [6,[15], [16], [17], [18]], but one study specifically identified disparities in patient portal completion for Black patients [8]. There were no adjusted differences for Hispanic/Latino individuals for response or response method in this study in contrast with past work [8,18], but the current findings for this subgroup should be interpreted with caution, given very limited representation of Hispanic patients in the overall sample. Patients who were not college graduates, especially those with an educational level of high school or less, were less likely to respond or to respond via email invitation alone. Similarly, a higher level of education was associated in past research with an increased likelihood of responding via an automated vs manual process at 1-year postoperatively across orthopaedic procedures [17]. There were no differences in response rate or response method for marital status, insurance status, or health literacy in the present study after adjusting for other factors. This contrasts with past studies that identified differences in response rates by marital status or health insurance, although not specifically at 1 year postoperatively [6,7,16]. We also did not identify differences in response rate or method by age despite past findings of such associations [6,[15], [16], [17],^19^,^20^]. This finding aligns with evidence that older adults have rapidly adopted digital tools for managing care, and doubts about their ability to use technology should not hinder tech-based PROM implementation [21]. However, we note that in our study, PROMs were completed electronically through secure, Health Insurance Portability and Accountability Act-compliant electronic platforms (DatStat and REDCap), accessed via personalized email links that went directly to the survey without requiring a participant log in password. There may have been other findings for age if using patient portals that require more complex log-in processes, unique passwords, and navigation to the survey.

Findings specific to factors such as race and education highlight systemic inequities that may hinder the completion of PROMs and limit the representation of patients within quality measures such as that mandated by CMS [3]. While electronic platforms enhance the generalizability and scalability of PROM collection, they also present unique barriers to equitable implementation. Patients from lower socioeconomic backgrounds—or those without access to compatible devices or reliable internet—may be unable to complete PROMs electronically. These digital limitations can contribute to lower response rates and should be considered when designing inclusive data collection strategies. Hospitals that predominantly serve marginalized communities may struggle to meet CMS reporting thresholds, thereby risking reduced funding, and further exacerbating existing inequities in health-care resources [3]. For example, 1 safety-net hospital reported a low PROM completion rate (∼18%) for THA and TKA patients at only 3 months with an automated PROM collection system [22].

For clinical factors, those who were cigarette smokers were less likely to respond overall, but no difference was observed for response method. Those with the highest comorbidity burden were less likely to respond following email invitation alone only in the adjusted analyses. Interestingly, there was no difference in musculoskeletal-specific comorbidities for either response or response method in the unadjusted analyses, and there were minimal to no differences in the adjusted analyses. The finding of an increased likelihood of responding for TKA vs THA patients has also been noted in a mixed sample of patients responding to preoperative and/or postoperative PROMs [7]. Generally, patients with better preoperative patient-reported health, such as less pain, better function, and better global mental health, were significantly more likely to respond to PROMs overall and following email invitation alone in unadjusted analyses. Similarly, another study identified increased response via an automated method at 1-year follow-up in those with better mental health [17]. However, the differences in preoperative mental health, pain, and function scores may not be clinically meaningful in this study as they were quite small and were primarily not present in the multivariable models. While patients with better preoperative health may have more capacity to engage in postoperative care and follow-up, in these analyses, sociodemographic factors played more of a role in PROM response and response method.

Finally, it is important to note that patients who reported reviewing the PROM data at their initial visit with their surgeon were more likely to respond without staff contact or support at 12 months. This observation suggests that when patients understand the value of the PROM data to their surgeon and their care, they are more likely to respond independently over time. Further research is needed to clarify strategies to engage patients and clinicians to use PROMs in the clinical encounter, as use may be associated with patient engagement with longitudinal reporting [23].

In the current study, average 1-year PROM mean scores did not significantly vary by method of response, aligning with past findings of similar outcomes with automated vs manual follow-up [17]. Continued research is needed to better understand bias in performance that may be introduced by nonrepresentative samples to balance the expenses of manual collection efforts with advantages in measurement accuracy. There may be solutions to address structural and individual barriers that hinder PROM completion that are less burdensome than added personnel effort [20,24]. Personalized follow-up strategies that are culturally sensitive and tailored may improve participation in the collection of patient-reported data [25]. These processes may include offering multilingual resources, patient education on the relevance of PROMs, and hybrid communication models. Designing meaningful solutions necessitates a deeper understanding of existing personal and structural barriers. For example, are barriers to independent completion following email invitation alone related to poor digital literacy, distrust of technology, or absence of relational communication that occurs via telephone? Different barriers require different solutions.

### Limitations

The study’s major strengths are its large multicenter sample and adjusted analyses, which controlled for confounding variables. However, the study also has several limitations that warrant consideration. First, the sample only included those who were eligible to participate in the trial after completing the preconsultation PROMs and who consented to participate in the trial, which may not have included those with the greatest risk of nonresponse at 12 months postsurgery. However, we note that for quality purposes, understanding nonresponse at follow-up specifically among those who responded at baseline is highly relevant given the CMS expectation for paired data. Most trial participants were English-speaking, which did not allow us to examine disparities in PROM completion among non–English-speaking populations, which have been identified in past work [6,7]. Second, this study did not specifically examine the role of digital or technological literacy or other cultural barriers, which may have contributed to the observed disparities in PROM response or response method [26]. We had limited information on patient socioeconomic status beyond Medicaid eligibility, such as income or area-level social deprivation, which have been previously associated with decreased response [7,18]. Also, it is important to note that the interplay of social determinants of health poses challenges in isolating the specific contributions of individual factors to PROM response rates. While the email invitation alone group served as a proxy for independent completion via electronic health record patient portals, patients in the trial received a direct personalized link to their survey. These findings may not generalize to typical electronic health record patient portal use, where participants typically must log in and navigate to the survey within the portal. However, digital commercial solutions increasingly utilize direct links with validation using identifiers such as name and date of birth vs an account log-in. Finally, while staff contact attempts were tracked for operational reasons during the study period, the average number of attempts required per patient was not calculated. As such, it is difficult to quantify the intensity of follow-up needed for PROM completion. Future work may benefit from analyzing contact frequency to better inform resource allocation for longitudinal PROM collection efforts.

## Conclusions

This study demonstrated that PROM response rates at 12 months after surgery and methods to support completion are associated with sociodemographic and clinical factors. From a health equity lens, Black patients were less likely to respond to PROMs following email invitation alone compared to White patients, and those with lower education were less likely to respond following email invitation or to respond at all compared to college graduates. These findings underscore the importance of hybrid approaches to engage patients to improve equity in postoperative PROM collection. Future research should explore the roles of digital literacy, language, and cultural factors in PROM completion and evaluate strategies to enhance engagement among diverse patient populations. Ensuring inclusivity in PROM data collection is essential for equitable healthcare delivery and improved health outcomes.

## Funding

Research reported in this article was funded through a 10.13039/100006093Patient-Centered Outcomes Research Institute Award (Grant No. IHS-1507–31714-IC, PI: Franklin). The statements in this article are solely the responsibility of the authors and do not necessarily represent the views of the Patient-Centered Outcomes Research Institute, its Board of Governors or Methodology Committee. Sylla’s time was funded by a T35 Summer Research Program for Medical Students (T35DK126628, PI: Lander) from the 10.13039/100000002National Institutes of Health. The content is solely the responsibility of the authors and does not necessarily represent the official views of the National Institutes of Health.

## Conflicts of interest

Brocha Z. Stern Stern is a committee member for the PROMIS Health Organization.

Linda I. Suleiman is a speaker for Zimmer Biomet, is a paid consultant for Zimmer Biomet, receives research funding from Zimmer Biomet, is on the editorial board for AAHKS Women in Arthroplasty and the Ruth Jackson Orthopaedic Society, and is a board/committee member for the Ruth Jackson Orthopaedic Society.

Patricia D. Franklin is an Associate Editor for the Journal of Advances in Patient Reported Outcomes and a member of the Arthritis Foundation Medical and Science Advisory Committee.

The other authors declare no potential conflicts of interest.

For full disclosure statements refer to https://doi.org/10.1016/j.artd.2025.101804.

## CRediT authorship contribution statement

**Fatoumata Sylla:** Writing – review & editing, Writing – original draft, Conceptualization. **Brocha Z. Stern:** Writing – review & editing, Supervision, Project administration, Conceptualization. **Martha Burla:** Writing – review & editing, Resources, Project administration, Conceptualization. **Linda I. Suleiman:** Writing – review & editing. **Patricia D. Franklin:** Writing – review & editing, Project administration, Methodology, Investigation, Funding acquisition, Conceptualization.

## Appendix A Supplementary data

Conflict of Interest Statement for Suleiman

Conflict of Interest Statement for Stern

Conflict of Interest Statement for Burla

Conflict of Interest Statement for Franklin

Conflict of Interest Statement for Sylla
