# Supplementary material for: Sociodemographic and Clinical Factors Associated With Response to 1-Year Postarthroplasty Patient-Reported Outcome Measures
Source: Arthroplast Today. 2026 Mar 30;37(Suppl):101804. doi: 10.1016/j.artd.2025.101804 (PMC13081220; doi:10.1016/j.artd.2025.101804)
Supplement: Conflict of Interest Statement for Stern [file mmc2.pdf]

# CONFLICT OF INTEREST STATEMENT

## *American Association of Hip and Knee Surgeons*

(Adopted from the American Academy of Orthopaedic Surgeons disclosure statement)

The following form **must be filled out completely and submitted by each author (example, 6 authors, 6 forms). All items require a response. If there is no relevant disclosure for a given item, enter "*None*."**

---

Manuscript Title
Sociodemographic and clinical factors associated with response to one year post-arthroplasty patient-reported outcome measures

1. Royalties from a company or supplier (The following conflicts were disclosed)
   None

2. Speakers bureau/paid presentations for a company or supplier (The following conflicts were disclosed)
   None

3A. Paid employee for a company or supplier (The following conflicts were disclosed)
   None

3B. Paid consultant for a company or supplier (The following conflicts were disclosed)
   None

3C. Unpaid consultants for a company or supplier (The following conflicts were disclosed)
   None

4. Stock or stock options in a company or supplier (The following conflicts were disclosed)
   None

5. Research support from a company or supplier as a Principal Investigator (The following conflicts were disclosed)
   None

6. Other financial or material support from a company or supplier (The following conflicts were disclosed)
   None

7. Royalties, financial or material support from publishers (The following conflicts were disclosed)
   None

8. Medical/Orthopaedic publications editorial/governing board (The following conflicts were disclosed)
   None

9. Board member/committee appointments for a society (The following conflicts were disclosed)
   PROMIS Health Organization, Committee chair (unpaid)

**Each author must sign AND print or type his/her name, date and submit a separate form**

In addition, one BLINDED Conflict of Interest form (no author names used) should be submitted per manuscript with all author disclosures.

| Brocha Stern | Brocha Z Stern Digitally signed by Brocha Z Stern Date: 2025.01.27 13:20:38 -05'00' | 01/27/2025 |
|---|---|---|
| Author Name (Print or Type) | Author Signature | Date |
